# The Influence of Random Element Displacement on DOA Estimates Obtained with (Khatri–Rao-)Root-MUSIC

**DOI:** 10.3390/s141121258

**Published:** 2014-11-11

**Authors:** Veronique Inghelbrecht, Jo Verhaevert, Tanja van Hecke, Hendrik Rogier

**Affiliations:** 1 Department of Industrial Technology and Construction, Ghent University, Valentin Vaerwyckweg 1, 9000 Gent, Belgium; E-Mails: Jo.Verhaevert@UGent.be (J.V.); Tanja.Vanhecke@UGent.be (T.H.); 2 Department of Information Technology, Ghent University, Sint-Pietersnieuwstraat 41, 9000 Gent, Belgium; E-Mail: Hendrik.Rogier@UGent.be

**Keywords:** DOA-estimation, Khatri-Rao, steering vector error, stochastic collocation method

## Abstract

Although a wide range of direction of arrival (DOA) estimation algorithms has been described for a diverse range of array configurations, no specific stochastic analysis framework has been established to assess the probability density function of the error on DOA estimates due to random errors in the array geometry. Therefore, we propose a stochastic collocation method that relies on a generalized polynomial chaos expansion to connect the statistical distribution of random position errors to the resulting distribution of the DOA estimates. We apply this technique to the conventional root-MUSIC and the Khatri-Rao-root-MUSIC methods. According to Monte-Carlo simulations, this novel approach yields a speedup by a factor of more than 100 in terms of CPU-time for a one-dimensional case and by a factor of 56 for a two-dimensional case.

## 1. Introduction

DOA estimation is a major application of the sensor array, since there are many real-world problems where an accurate estimate of the source direction is essential: for example, in radar, electroencephalogram, sonar and microphone array systems. The number of sources that can be resolved by an *N*-element uniform linear array using traditional subspace-based methods, such as MUSIC, is *N* − 1. Recently, more advanced methods have been presented for DOA estimation, for example, the Khatri–Rao (KR) product approach. By assuming quasi-stationary sources, this concept can identify up to 2*N* − 1 sources using an *N*-element uniform linear array, without computing higher-order statistics [1].

Irrespective of the DOA estimation algorithm applied, deviations between the actual steering vector and the presumed steering vector are unavoidable in most applications that rely on an array of sensors. Errors during fabrication, uncalibrated arrays and arrays undergoing deformations are among the potential factors that can contribute to such errors. Moreover, the arrays may be composed of sensor elements with directional radiation patterns [2–4]. In most papers on DOA estimation, the authors assume that there is no steering vector error or that steering vector deviations are deterministic and constant in time. This has resulted in the development of algorithms that estimate such errors and, subsequently, remove them by a calibration procedure [5–9]. In many cases, however, these errors are random and, therefore, statistical in nature. The motivation of our work is that most calibration methods are computationally expensive and/or time consuming. Moreover, to estimate the modeling errors in the array, often, certain assumptions have to be made, additional experiments have to be carried out and/or specific training sequences must be transmitted. Therefore, these techniques are difficult to apply in real-time applications or in systems where the random errors change quickly. Hence, it is interesting to take into account the effect of uncertainty in the placement of the sensor elements by means of a stochastic framework. Hence, there is a need for stochastic simulation tools that provide the statistical data to quantify the effect of the random displacement of these sensor elements on the DOA estimates.

Conventionally, one may use Monte-Carlo simulations to quantify the effect of position errors of one of the sensor elements to characterize the resulting distribution of the estimated DOAs. However, Monte-Carlo simulations require a large amount of realizations to accurately capture the statistics of the random process. Hence, the method is time consuming.

In this paper, we introduce the stochastic collocation method (SCM) [10] as a more effective way to rapidly model uncertainty in the estimated DOAs, due to variations in the elements of the steering vector. Thanks to this method, we can reduce the CPU time by a factor of more than 100 for a one-dimensional displacement and by a factor of 56 for a two-dimensional displacement of one of the sensor elements.

In this paper, we apply the nominal steering vector of a uniform linear sensor array to estimate the DOAs of a signal impinging on a sensor array where one of the sensors has a random position error. We establish a stochastic framework to find the probability density function (pdf) of the DOA-estimates. To the best of the authors' knowledge, such a framework has not been established in the open literature yet. In [11], a first-order sensitivity analysis is carried out to study the effect of modeling errors on the conventional MUSIC algorithm. This sensitivity analysis results in a sensitivity parameter and failure threshold, but does not provide the complete statistical distribution of the modeling errors. In [12], the authors examine a resolution threshold of the MUSIC algorithm for situations in which the array response is perturbed from its assumed value. This could be a boundary approximation for our framework. However, they assume that the arrays are illuminated with two equal power emitters.

In order to illustrate and validate the SCM method, we derive the statistical distribution on the DOA estimates for the root-MUSIC algorithm and for the KR-root-MUSIC algorithm. The latter algorithm applies the root-MUSIC method to the noise subspace matrix of the KR algorithm, as described in [1]. For the (KR)-root-MUSIC algorithm, we derive the cumulative distribution function (cdf), for one- and two-dimensional displacements of one of the sensor elements.

In earlier work, it has been proven that underdetermined DOA estimation is possible when the source signals are non-Gaussian stationary. In [13], by the use of fourth-order cumulants, a virtual array with an increase in the degrees of freedom is achieved. In [14], a new array geometry, which is capable of increasing the degrees of freedom of linear arrays, is proposed. The structure is obtained by systematically nesting two or more uniform linear arrays. It can provide *N*^2^ degrees of freedom using only *N* physical sensors and the second-order statistics of the data. Blind source separation, by use of quasi-stationarity, has also received attention. One technique is based on the least squares fitting (LSF) criterion, using parallel factor analysis (PARAFAC) [15]. One can prove that quasi-stationarity enables the identification of sources when the number of sensors is lower than the number of sources. However, the LSF is based on a multi-dimensional non-linear optimization problem. In [16], the authors have used a method called focusing Khatri–Rao subspace (FKR) for wideband array processing. They calculate a focusing matrix using a rotational signal-subspace [17], and then, the covariance matrices of different frequencies are transformed and combined by means of the KR-product. Recently, in [18], the authors used sparse covariance fitting in order to estimate underdetermined DOA estimation, and in [19], a sparse representation of the array covariance vector is used to obtain the DOAs. In [20,21], the Khatri–Rao approach is also extended to a uniform circular array. In [22], the Khatri–Rao approach is used for 2D DOA-estimation. In [23], the authors make use of a maximum likelihood method to obtain the underestimated DOAs for a multiple-input multiple-output radar.

Notations: We denote matrices and vectors by boldfaced capital letters and lower-case letters, respectively. Superscript*^H^* denotes the transpose conjugate, whereas superscript*^T^* denotes the transpose without the conjugate. For a given vector **x** ∈ ℂ, ‖x‖ denotes its Euclidean norm. For a given matrix A ∈ ℂ*^M^*^×^*^N^*, the *i*-th column is denoted by a_i_. The notation *vec*(**A**) stands for vectorization; *i.e.*, if **A** = [a_1_,…, a_N_], then *vec*(**A**) = [a_1_*^T^*,…, a_N_*^T^*]*^T^*.

The remainder of the paper is organized as follows. In Section 2, we present the signal model and the KR-MUSIC algorithm [1]. Section 3 describes how the steering vector and the manifold array change if there is a displacement in one of the sensor elements. A brief description of the SCM, which is applied to model the uncertainty due to statistical variations of the input parameters, is given in Section 4. The proposed method is validated in Section 5, and conclusions are drawn in the last section.

## 2. Signal Model and Summary of the KR-MUSIC Algorithm

In this section, we give a short summary of the KR-MUSIC algorithm (see [1]), preceded by the signal model used.

### 2.1. Signal Model

Consider *K* sources impinging on a uniform linear sensor array, consisting of *N* omnidirectional sensors and neglecting mutual coupling. For a signal *S_k_*(*t*) emitted by the *k*-th source, let s(*t*) = [*s*_1_(*t*),…,*s_K_*(*t*)]*^T^* denote the *K* × 1 source signal vector. Let $\text{a}(\theta )={\left[1,e^{j2\pi \frac{d}{\lambda }\sin(\theta )},\ldots ,e^{j2\pi \frac{d}{\lambda }(N-1)\sin(\theta )}\right]}^{T}$ be the *N* × 1 steering vector corresponding to the direction *θ*, with *d*, the spacing between the *N* sensors and λ the signal wavelength. In this paper, we fix the element spacing to *d*/λ = 1/2. Our aim is to study the effect of random variations in the array geometry on algorithms estimating the directions of arrival *θ_k_* corresponding to the K source signals, in the presence of measurement noise. Therefore, **A** = [a(*θ*_1_) a(*θ*_2_) … a(*θ_k_*)] describes the *N* × *K* array manifold matrix and v(*t*) = [*υ*_1_(*t*),…, *υ_N_*(*t*)]*^T^* ∈ ℂ*^N^*^×1^ represents the spatial and temporal white noise. For the observed signal *x_n_*(*t*) of the *n*-th sensor, let **x**(*t*) = [*x*_1_(*t*),…, *x_N_*(*t*)]*^T^* denote the *N* × 1 receiver signal vector. This vector equals:

(1) $$\begin{array}{cc} \text{x}\left(t\right)=\text{As}\left(t\right)+\text{v}\left(t\right), & t=0,1,2,\ldots \end{array}$$

The DOA-estimation algorithms under study are the root-MUSC and KR-root-MUSIC algorithms. These algorithms rely on the following assumptions:

- The noise **v**(*t*) is white Gaussian and zero-mean wide-sense stationary with covariance matrix C ≜ *E*{‖**v**(*t*)‖^2^}. It is statistically independent of the source signals.
- All source DOAs are distinct: *θ_k_* ≠ *θ_l_* for *k* ≠ *l*.
- The source signals *s_k_*(*t*) are mutually uncorrected and have a zero mean.
- Each source signal is wide-sense quasi-stationary with frame length *L*, such that:
  (2) $$\begin{array}{ccc} E\left\{{\left\|s_{k}\left(t\right)\right\|}^{2}\right\}=d_{m,k}, & \forall t\in \left[\left(m-1\right)L,mL-1\right], & m=1,2,\ldots \end{array}$$
  with *d_m,k_* the frame-dependent and sensor-dependent average normalized signal power. This assumption means that the second-order statistics of the source signals are time varying, but that they remain static over a short period of time. Under this quasi-stationarity assumption, we define a local covariance matrix:
  (3) $$\begin{array}{l} {\text{R}}_{\text{m}}=E\left\{{\left\|x\left(t\right)\right\|}^{2}\right\}\in {\text{C}}^{N\times N},\\ \forall t\in \left[\left(m-1\right)L,mL-1\right],m=1,2,\ldots \end{array}$$

In the remainder of the paper, we apply the DOA estimation algorithms to synthetic signals generated by the procedure proposed in [1] to construct source signals *s_k_*(*t*) satisfying the above constraints, with sequence length *T* = 25,600 and an allowable range of the frame periods [*L_low_*, *L_u_*_pp_] = [300,700]. Algorithm 1 is applied to generate the synthetic signals.



Set *T_cur_* = 0. *T_cur_* is the start of a new frame of the signal;**while**
*T_cur_* < *T*
**do** Randomly generate *L_f_* following a uniform distribution on [*L_low_*, *L_upp_*]; Randomly generate *σ_s_* following a uniform distribution on [0,1]; **for**
*t* = *T_cur_*
**to**
*T_cur_* + *L_f_*− 1 **do**  Randomly generate *S_k_*(*t*) = *s_R_*(*t*) + *js_I_*(*t*), where *s_R_*(*t*) and *s_I_*(*t*) are independent and identically Laplacian distributed with zero mean and variance 
$\sigma_{\text{s}}^{2}/2$ **end** *T_cur_* := *T_cur_* + *L_f_*;  **end****Algorithm 1:** The algorithm to generate synthetic signals.


Observe that the frame intervals of the different impinging sources are not fixed and not synchronized. We apply the KR subspace methods by choosing a fixed frame period of *L* = 512. The number of frames is set to *M* = *T*/512 = 50.

### 2.2. Summary of the KR-MUSIC Algorithm

For a given receiver signal vector ${\left\{X(t)\right\}}_{t=0}^{T-1}$, with frame length *L*, where *L* divides *T*, the KR-MUSIC algorithm proceeds as follows [1]:

- Compute the local variance estimates ${\hat{\text{R}}}_{m}=\frac{1}{L}\overset{mL-1}{\underset{t=(m-1)L}{\text{∑}}}\text{x}(t){\text{x}}^{H}(t)\;\text{for}\;m=1\ldots M$.
- Denote an orthogonal complement projector ${\text{P}}_{1\text{M}}^{⊥}={\text{I}}_{\text{M}}-\frac{1}{M}1_{\text{M}}{1_{\text{M}}}^{T}$, with I_M_ the *M* × *M* identity matrix and 1_m_ the *M* × 1 all-one vector. Perform the projection $\bar{\text{Y}}={\hat{\text{Y}}\text{P}}_{1\text{M}}^{⊥}(\bar{\text{Y}}\in {\text{ℂ}}^{N^{2}=M})$, with **Ŷ** the data matrix formed by vectorize the covariance matrix. This projection eliminates the unknown noise covariance.
- As in [1], perform a dimension reduction on **Y̅** obtaining **Ỹ**.
- To apply MUSIC to **Ỹ**, perform a singular value decomposition (SVD; on:
  (4) $$\tilde{\text{Y}}=\text{U}\Sigma {\text{V}}^{H}$$
  where **U** ∊ ℂ ^(2^*^N^*^−1)×^*^k^* and **V** ∊ ℂ*^M^*^×^*^K^* are the left and right singular matrices, respectively, and **Σ** ∊ ℝ*^K^*^×^*^K^* is the diagonal singular values matrix with the singular values being arranged in descending order.
- By extracting the noise subspace matrix **U_n_** = [**u_K+1_**, …, **u_2N−1_**] ∈ ℂ^(2^*^N^*^−1)×(2^*^N^*^−1−^*^K^*^)^from **U**, we compute the DOA spectrum:
  (5) $$P_{\text{KR}-\text{MUSIC}}\left(\theta \right)=\frac{1}{{\left\|{{\text{U}}_{\text{n}}}^{H}{\text{W}}^{\frac{1}{2}}\text{b}\left(\theta \right)\right\|}^{2}}$$

Define **b_r_**(*z*) ∊ ℂ^1×(2^*^N^* ^− 1)^ as:

(6) $${\text{b}}_{\text{r}}\left(z\right)={\left[z^{-\left(N-1\right)},\ldots ,z^{-1},1,z,z^{2},\ldots .z^{N-1}\right]}^{T}$$

and $\text{b}(\theta )={\text{b}}_{\text{r}}(e^{j2\pi \frac{d}{\lambda }\sin(\theta )})$. The *K* largest local maxima of the DOA spectrum *P*_KR-MUSIC_(*θ*) are the DOA estimates.

Instead of searching the peaks of the MUSIC spectrum, we can also apply the root-MUSIC method [24] to ‖**U**_**n**_*^H^***W**‖^2^. In the remainder of the paper, we call the resulting method the KR-root-MUSIC method.

## 3. Displacement of One of the Sensor Elements

**Figure 1.**
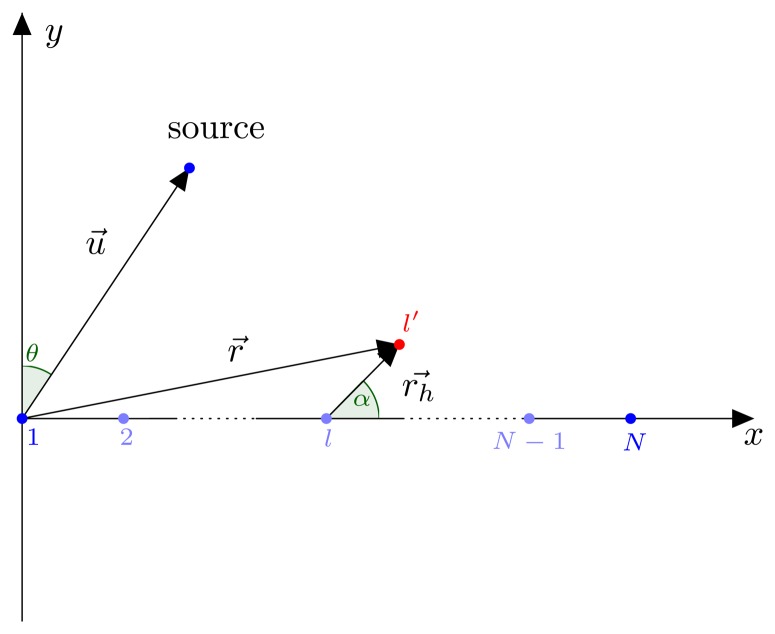
Displacement of the *l*-th sensor element.

In this section, we assume that one of the sensor elements is displaced due to an unintentional movement of an element. Figure 1 depicts an array of *N* sensor elements, where the first sensor element is placed at the origin of the *xy*-plane and the other elements along the *x*-axis of this plane. The *l*-th sensor element is displaced by a vector $\vec{r_{h}}$ with respect to its original position.

Denote $r_{h}=||\vec{r_{h}}||$ and $\alpha =\arg(\vec{r_{h}})$. The new coordinates of the displaced *l*-th sensor are given by *r⃗* = [(*l* − 1)*d* + *r_h_* cos(*α*), *r_h_* sin(*α*)]. Define *β* = arg(*r⃗*) and *r* = ‖*r⃗*‖, such that $\frac{2\pi }{\lambda }\vec{u}\cdot \vec{r}=\frac{2\pi }{\lambda }r\sin(\theta +\beta )$. *u⃗* is the unit vector with the same direction of the position vector of the source, which is located in the array's far field. The *l*-th row of the array manifold matrix then becomes $\left[e^{j2\pi \frac{r}{\lambda }\sin(\theta_{1}+\beta )},e^{j2\pi \frac{r}{\lambda }\sin(\theta_{2}+\beta )},e^{j2\pi \frac{r}{\lambda }\sin(\theta_{K}+\beta )}\right]$.

## 4. Stochastic Collocation Method

In order to investigate the influence of a stochastic displacement of one of the sensor elements, we need stochastic simulation tools to characterize the statistical distribution of the DOAs due to the distribution of the random displacement vectors.

A Monte-Carlo simulation for such a task requires a large amount of realizations to accurately capture the statistics of the random process. Therefore, we apply the SCM proposed in [10] as a more effective way to rapidly model geometrical uncertainty, due to random variations of the array element positions.

### 4.1. One-Dimensional Stochastic Input

Assume that an input random variable *X* (in our case, e.g., the displacement in the *x*- or *y*-direction) is given. We want to determine the variation of the output *Y* = *f*(*X*) (in this paper, the DOA estimates), due to the statistical variation of *X*. The random variable *X* follows the cumulative distribution *P^X^* and probability density function *dP^X^* in the sample space Ω. To determine the statistics of *Y*, we rely on the Askey scheme [25] to approximate the transformation *Y* = *f*(*X*) by a polynomial expansion of order *P*.

(7) $$Y\approx f^{P}(X)=\sum_{k=0}^{P}y_{k}^{X}\phi_{k}^{X}(X),$$

with $\phi_{k}^{X}(X)$ the expansion polynomials and $y_{k}^{X}$ the weights belonging to the *k*-th expansion polynomial. *f^P^*(*X*) is the generalized polynomial chaos expansion (GPoC expansion) of *Y* = *f*(*X*).

An optimal expansion is obtained when the set of expansion polynomials forms a complete orthogonal basis in Ω with orthogonality relation:

(8) $$\begin{array}{c} \langle \phi_{i}^{X}(x),\phi_{j}^{X}(x)\rangle =\int_{\Omega }\phi_{i}^{X}(x)\phi_{j}^{X}(x)dP^{X}\left(x\right)\\ =\langle \left(\phi_{i}^{X}{\left(x\right)}^{2}\right)\rangle \delta_{ij}, \end{array}$$

with the Kronecker *δ_ij_* = 0 if *i* ≠ *j* and *δ_ij_* = 1 if *i* = *j*.

In this case, the Cameron-Martin [26] convergence theorem ensures exponential convergence to the function *Y* = *f*(*X*) for *P* → ∞. In the remainder of the paper, we assume that the statistical variation of *X* follows a Gaussian distribution in the sample space Ω. Relying on the Askey scheme, we know that the probabilistic Hermite polynomials *H_k_*(*X*) provide an optimal expansion for the normal Gaussian distribution [25].

To determine the unknown expansion coefficients $y_{k}^{X}$, we apply Galerkin weighting to Equation (7) [27]:

(9) $$y_{k}^{X}=E\left[Y\left(x\right)\phi_{k}^{X}\left(x\right)\right]=\int_{\Omega }Y\left(x\right)\phi_{k}^{X}(x)dP^{X}\left(x\right)$$

We can approximate this integral by a *Q*-point Gauss-Hermite quadrature rule, being [28]:

(10) $$\begin{array}{cc} y_{k}^{X}\approx \overset{Q}{\underset{i=1}{\text{∑}}}w_{i}Y\left(x_{i}\right)\phi_{k}^{X}(x) & k=0,1,\ldots ,P. \end{array}$$

where the quadrature points *x_i_* are given by the *Q* zeros of $\phi_{Q}^{X}(x)$ in Ω and with *w_i_* the corresponding weights. In order to evaluate Equation (10), we must determine *Y* = *f*(*X*) for *Q* realizations of the random variable *X*, corresponding to the quadrature points. When utilizing a Gaussian quadrature, Equation (10) exactly integrates all polynomials of degree equal or less than 2*Q* − 1. Given an expansion order *P*, the highest order coefficient evaluation of Equation (9) can be assumed to involve integrands of at least polynomial order 2*P*, being *ϕ^X^*(*x*) of order *P* and *Y*(*x*) modeled to order *P*, such that a minimal Gaussian quadrature order of *P*+1 will be required to obtain good accuracy in these coefficients. Given the exponential convergence of the expansion (7), using the SCM theory, we need to apply the KR-root-MUSIC algorithm in the *Q* = *P* + 1 quadrature points. The Monte-Carlo analysis may then be applied to the computationally cheap expansion (7) instead of to the KR-root-MUSIC. This results in huge savings in CPU-time, as the Monte-Carlo method is only slowly converging with respect to the number of samples taken, being inversely proportional to the square root of the number of samples [29].

### 4.2. Two-Dimensional Stochastic Input

We assume that the variation of the output *Y* = *f*(*X*_1_*, X*_2_) depends on two independent stochastic variables *X*_1_ and *X*_2_, respectively, in the sample space Ω_1_ and Ω_2_, respectively (e.g., a displacement in the *x*- and *y*-direction).

We first extend the GPoC expansion (7) to two dimensions. For *Y* = *f*(*X*_1_, *X*_2_), we obtain:

(11) $$Y=f\left(X_{1},X_{2}\right)=\sum_{i=0}^{P_{1}}\sum_{j=0}^{P_{2}}y_{ij}^{X_{1}X_{2}}\phi_{i}^{X_{1}}\left(X_{1}\right)\phi_{j}^{X_{2}}\left(X_{2}\right)$$

which is a polynomial expansion of degree *P*_1_ in *X*_1_ and *P*_2_ in *X*_2_. The weight coefficients are given by:

(12) $$y_{ij}^{X_{1}X_{2}}=\int_{\Omega_{1}}\int_{\Omega_{2}}Y\left(x_{1},x_{2}\right)\phi_{i}^{X_{1}}\left(x_{1}\right)\phi_{j}^{X_{2}}\left(x_{2}\right)dP^{X_{1}}\left(x_{1}\right)dP^{X_{2}}\left(x_{2}\right)$$

or if we approximate Equation (12) by a tensor product quadrature rule:

(13) $$y_{ij}^{X_{1}X_{2}}=\sum_{k=1}^{Q_{1}}\sum_{l=1}^{Q_{2}}w_{k}\phi_{i}^{X_{1}}\left(u_{k}\right)f\left(u_{k},u_{l}\right)w_{l}\phi_{j}^{X_{2}}\left(u_{l}\right),$$

with the quadrature points *u_k_* and *u_l_* given by the *Q*_1_ zeros of $\phi_{Q_{1}}^{X_{1}}(x_{1})$ and the *Q*_2_ zeros of $\phi_{Q_{2}}^{X_{2}}(x_{2})$, respectively. Equation (13) requires *Q*_1_ · *Q*_2_ function evaluations. If we only need a small number of quadrature points (whenever we can model *Y* as a polynomial of low degree), this is a very effective numerical tool. However, the number of function evaluations grows quadratically with the number of quadrature points *Q* (in the case that *Q* = *Q*_1_ = *Q*_2_).

In the case that *X*_1_ and *X*_2_ are independent Gaussian distributed variables with zero mean and dispersions *σ*_1_ and *σ*_2_, respectively, we can rewrite Equation (12) as:

(14) $$\begin{array}{c} y_{i,j}^{X_{1}X_{2}}=\int_{\Omega_{1}}\int_{\Omega_{2}}\frac{e^{-x_{1}^{2}-x_{2}^{2}}}{\pi }H_{i}(\sqrt{2}\sigma_{1}x_{1})H_{j}(\sqrt{2}\sigma_{2}x_{2})\\ \cdot Y(\sqrt{2}\sigma_{1}x_{1},\sqrt{2}\sigma_{2}x_{2})dx_{1}dx_{2}\\ =\int_{\Omega_{1}}\int_{\Omega_{2}}p(x_{1},x_{2})e^{-x_{1}^{2}-x_{2}^{2}}dx_{1}dx_{2}, \end{array}$$

with,

(15) $$p(x_{1},x_{2})=\frac{1}{\pi }H_{i}(\sqrt{2}\sigma_{1}x_{1})H_{j}(\sqrt{2}\sigma_{2}x_{2})Y(\sqrt{2}\sigma_{1}x_{1},\sqrt{2}\sigma_{2}x_{2}).$$

We approximate this integral by using cubature formulas for the plane with weight function *e*−*^r^*^^2^^ ([30,31]):

(16) $$y_{i,j}^{X_{1},X_{2}}=\sum_{k=1}^{Q}w_{k}p(x_{1,k},x_{2,k}),$$

with (*x*_1,_*_k_*, *X*_2,_*_k_*) the quadrature points and *W_k_* the corresponding weights. For these formulas, the number of function evaluations is limited to *Q*. We call *d_e_* the degree of the cubature formula, meaning that Equation (16) is exact for polynomials of degree *d_e_*.

The cubature formulas that we have applied in this paper are those from [32] with *Q* = 44, *d_e_* = 15, those from [33] with *Q* = 99, *d_e_* = 21 and those from [33] with *Q* = 172, *d_e_* = 31.

Given the exponential convergence of the polynomial expansions, for the two-dimensional expansion, we have to apply the KR-root-MUSIC algorithm only *Q*_1_ · *Q*_2_ times, when making use of the tensor rule Equation (13). Whereas, if we use the cubature formulas following Equation (16), we need to apply the KR-root-MUSIC algorithm *Q* times. Once we have estimated the DOAs in the quadrature points, the whole distribution can be obtained by applying the Monte-Carlo method to the computationally cheap polynomial expansion instead of to the DOA estimation algorithms.

## 5. Results

In all simulation examples below, the signal-to-noise ratio SNR (in dB) is denned as:

(17) $$\text{SNR}=10{\text{log}}_{10}\left(\frac{E\left\{{\left\|\text{As}(t)\right\|}^{2}\right\}}{E\left\{{\left\|\text{v}(t)\right\|}^{2}\right\}}\right)$$

In order to validate the efficiency of the SCM method, we will provide several numerical examples. We first consider the case where the displacement vector $\vec{r_{h}}$ is a constant vector. Next, we focus on the more general case where the *x*- and/or the *y*-coordinates of the displacement vector are random variables.

In Section 5.1, we investigate the influence on the DOA estimates obtained by root-MUSIC and KR-root-MUSIC for a displacement of one sensor element, in absence of noise. In Section 5.2, we use the SCM method to approximate the curves obtained in Section 5.1. In Section 5.3, we analyze the cdf for a displacement of the sensor element in one direction, for noisy data signals. In Section 5.4, we derive the GPoC expansion by means of the SCM method for a two-dimensional shift.

### 5.1. One Shifted Sensor Element: DOA Estimation in Absence of Noise

Consider the case of *K* = 3 sources and *N* = 4 sensor elements. The true DOAs are assumed to be [−18°, 5°, 25°]. We apply the synthetic signal generation procedure of Section 2.1 to generate a random source signal and use this signal vector throughout the simulations. We furthermore assume that no noise has been added to the impinging signal. We assume that one of the sensor elements has moved along the *x*-axis. For $e_{x}^{\text{rel}}$, being the relative displacement (with respect to the distance *d* between two sensor elements) of the sensor element, $\vec{r_{h}}$ corresponds to coordinates [ $e_{x}^{\text{rel}}d$, 0].

**Figure 2.**
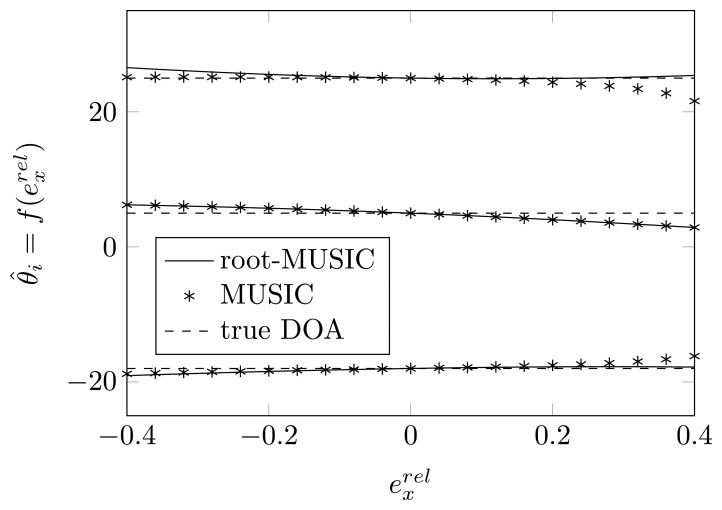
The estimated DOAs by means of (root)-MUSIC as a function of a shift of the first sensor element along the *x*-axis for true DOAs = [−18°, 5°, 25°] and (*N*, *K*) = (4, 3).

**Figure 3.**
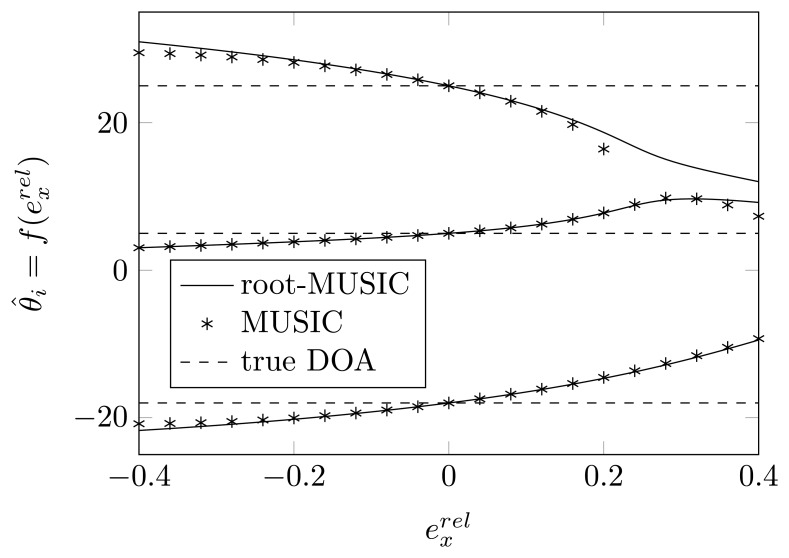
The estimated DOAs by means of (root)-MUSIC as a function of a shift of the second sensor element along the *x*-axis for true DOAs = [−18°, 5°, 25°] and (*N*, *K*) = (4,3).

In Figures 2 and 3, we plot the estimated DOAs, as a function of $e_{x}^{\text{rel}}$ for a relative displacement of each of the sensor elements for the root-MUSIC algorithm and the MUSIC algorithm. As a reference, the figures also show the true DOAs as dashed lines. We observe that even without any noise, the MUSIC algorithm is sensitive to a shift of one of the sensor elements and that if that shift is too large, in some cases, we do not find peaks in the MUSIC spectrum, such as in Figure 3 for $e_{x}^{\text{rel}}>0.2$. On the same figure, we observe that the root-MUSIC algorithm [24] estimates all DOAs, even in these cases where the conventional MUSIC algorithm does not find enough peaks. Furthermore, we observe that the DOAs estimated by the root-MUSIC algorithm and those estimated by the MUSIC algorithm are almost the same. The figures for a displacement of the first and fourth array element and those for a displacement of the second and third array element will almost be symmetric.

**Figure 4.**
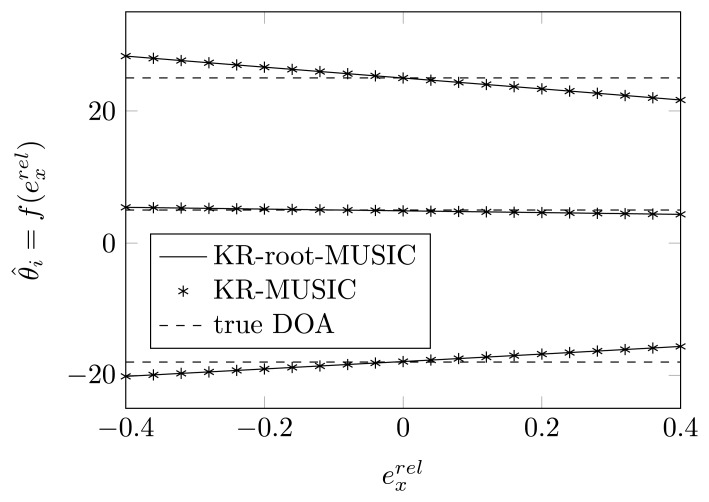
The estimated DOAs by means of Khatri-Rao (KR)-(root)-MUSIC as a function of a shift of the first sensor element along the *x*-axis for true DOAs = [−18°, 5°, 25°] and (*N*, *K*) = (4, 3).

**Figure 5.**
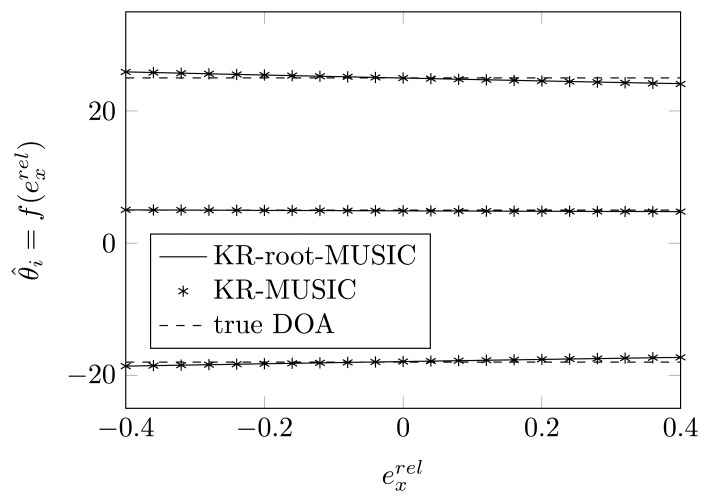
The estimated DOAs by means of KR-(root)-MUSIC as a function of a shift of the second sensor element along the *x*-axis for true DOAs = [−18°, 5°, 25°] and (*N*, *K*) = (4, 3).

In Figures 4 and 5, we have plotted the estimated DOAs, as a function of $e_{x}^{\text{rel}}$, for a random relative displacement of the first and second sensor element for the KR-MUSIC and KR-root-MUSIC algorithm. In contrast to MUSIC, the KR-MUSIC algorithm is more robust to shifts in one of the sensor elements. The Khatri-Rao transformation expands the dimension of the vector on which we perform the SVD and the extracted noise subspace matrix. This increases the probability of finding *K* peaks. We also observe that in the KR-MUSIC case, the estimated curves are almost linear as a function of the shifts. Furthermore, we observe that DOAs estimated by using the KR-MUSIC algorithm are almost the same as those estimated by the KR-root-MUSIC algorithm.

### 5.2. One-Dimensional GPoC Expansion

In this section, we start from the same assumptions as in Section 5.1, concerning the impinging signal and the sensor array.

**Figure 6.**
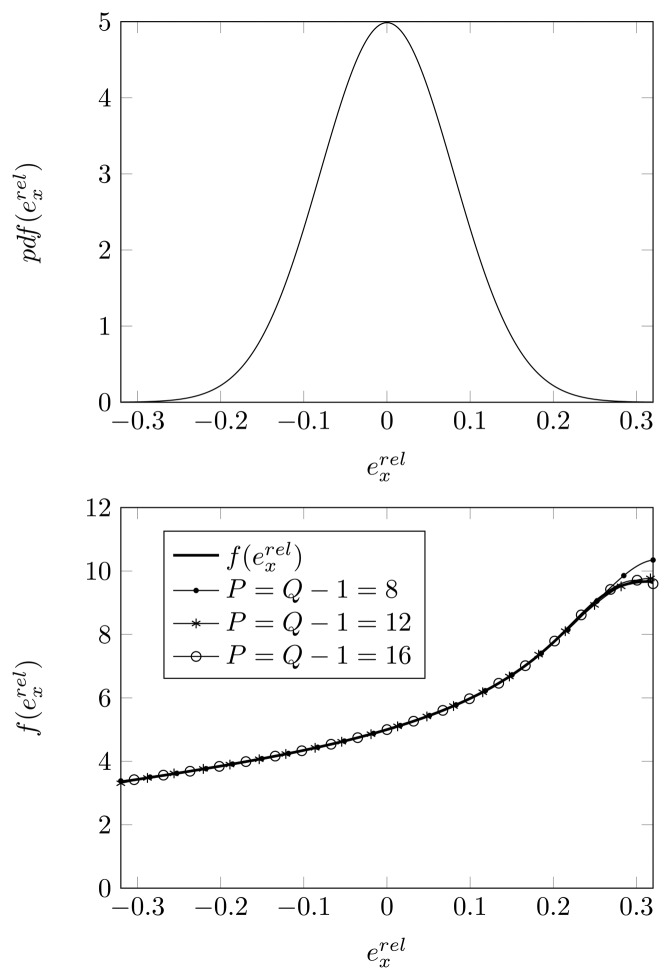
**(Top)** The Gaussian distribution with zero mean and *σ_pl_* = 0.08; **(Bottom)** root-MUSIC DOA estimate, approximated by the generalized polynomial chaos (GPoC) of order *P*, with *Q* quadrature points as a function of $e_{x}^{rel}$, for a displacement of the second sensor element, true DOA = 5° and (*N*, *K*) = (4, 3).

Let $f(e_{x}^{\text{rel}})$ be the estimation of the second DOA (true DOAs = [−18°, 5°, 25°]) as a function of the relative displacement of the second sensor element along the *x*-axis $e_{x}^{\text{rel}}$. As plotted in the top figure of Figure 6, the relative displacement $e_{x}^{\text{rel}}$ is assumed to be Gaussian distributed with dispersion *σ_pl_* = 0.08. As outlined in Section 4, we therefore apply Hermite polynomials in the GPoC expansion of $f(e_{x}^{\text{rel}})$. We use the SCM method (with *Q* quadrature points and a polynomial expansion of order *P* = *Q* − 1) to determine the expansion coefficients for the GPoC expansion. In the bottom figure of Figure 6, we have plotted the function $f(e_{x}^{\text{rel}})$ and the GPoC expansion of $f(e_{x}^{\text{rel}})$. We remark that, for $e_{x}^{\text{rel}}$ between −0.16 and 0.16, all plotted curves coincide within 1%. We also observe that, if we make use of a higher expansion order for the polynomials, the GPoC expansions better approximate the curve representing $f(e_{x}^{\text{rel}})$. For a polynomial expansion of order 16, the GPoC curve coincides with the $f(e_{x}^{\text{rel}})$ curve within 8% in the observed interval.

**Figure 7.**
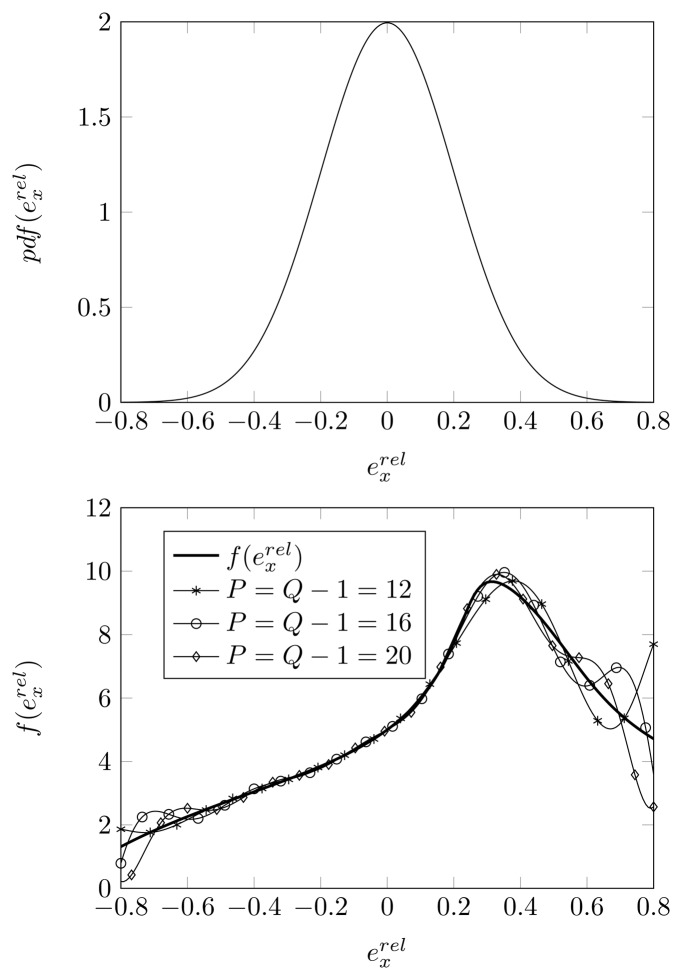
**(Top)** The Gaussian distribution with zero mean and *σ_pl_* = 0.2; **(Bottom)** root-MUSIC DOA estimate, approximated by the GPoC of order *P*, with *Q* quadrature points as a function of $e_{x}^{rel}$, for a displacement of the second sensor element, true DOA = 5° and (*N*, *K*) = (4, 3).

In Figure 7, we apply the SCM method with *P* = *Q* − 1 for $e_{x}^{\text{rel}}$ Gaussian distributed with dispersion *σ_pl_* = 0.2. We observe that, in the 95% confidence interval [−0.4,0.4] for $e_{x}^{\text{rel}}$, the GPoC based on the Hermite polynomials of order 12 or higher provides an accurate approximation for $f(e_{x}^{\text{rel}})$. However, beyond the 95% confidence interval, the Hermite polynomials rapidly oscillate, and they do not yield a good approximation for the $f(e_{x}^{\text{rel}})$ curve.

**Figure 8.**
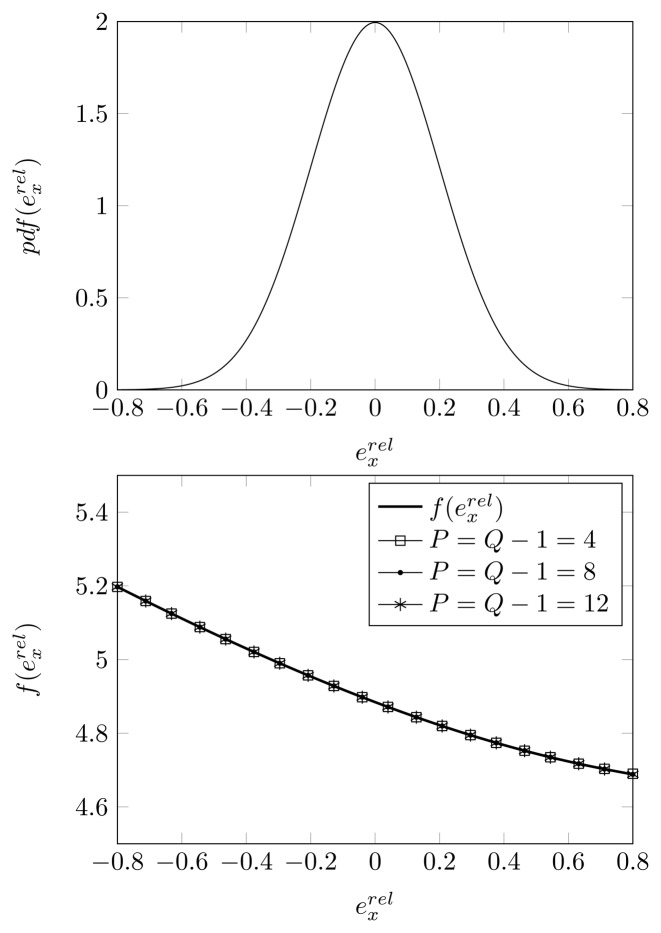
**(Top)** The Gaussian distribution with zero mean and *σ_pl_* = 0.2; **(Bottom)** KR-root-MUSIC DOA estimate, approximated by the GPoC of order *P*, with *Q* quadrature points as a function of $e_{x}^{rel}$, for a displacement of the second sensor element, true DOA = 5° and (*N*, *K*) = (4, 3).

In Figure 8, we have made the same assumptions as in Figure 7, but we have estimated the DOA using the KR-root-MUSIC algorithm. The curves coincide, even when restricting the GPoC to only *P* = 4.

### 5.3. One Shifted Element: Statistical Distribution of DOA Estimates

In this section, we again apply the synthetic signal generation procedure of Section 2.1 to generate a source signal. We construct an arbitrary complex Gaussian distributed noise vector **v**(*t*) = *υ_R_*(*t*) + *υ_I_*(*t*). *υ_R_*(*t*) and *υ_I_*(*t*) are both Gaussian distributed with zero mean and dispersion *σ* = 0.3 providing an SNR = 7.78 dB. Throughout the simulations, we use this source signal vector and noise vector. We also assume that the first sensor element is displaced along the *x*-axis and that the relative displacement $e_{x}^{\text{rel}}$ is Gaussian distributed with zero mean and dispersion *σ_pl_* = 0.12. To obtain the cdfs, we use two different methods: Monte-Carlo (MC) simulation and SCM. We carry out an MC simulation (where we vary $e_{x}^{\text{rel}}$) with 10,000 realizations. The CPU-time to carry out this simulation equals about 126 s for the root-MUSIC algorithm and 157 s for the KR-root-MUSIC algorithm. The simulations were performed on a Dell Latitude E6520PC with an Intel(R) Core(TM)i5-2520M 2.5 GHz processor and 4 GB RAM. The MATLAB version used is 8.0.0.783 (R2012b).

We also apply the SCM theory for different values of the quadrature points *Q* and a polynomial expansion of *Y* of order *P* = *Q* − 1. We perform a two-sample Kolmogorov–Smirnov test (KS test) [34], to compare the samples obtained by the MC method with those obtained by the SCM method. The two-sample KS test verifies the null hypothesis that the two samples come from the same distribution. The test statistic is denned as:

(18) $$D_{n,n^{\prime }}=\underset{x}{\sup}(\left|F_{1,n}(x)-F_{2,n^{\prime }}(x)\right|),$$

with *sup_x_*(*f*(*x*)) the supremum of the function *f*(*x*). *F*_1,_*_n_*(*x*) is the empirical cdf obtained with the MC and *F*_2,_*_n_*_′_(*x*) the empirical cdf obtained with SCM. *n* = *n′* = 10, 000 are the number of samples used for the MC and SCM method. The null hypothesis is rejected at level *α* if $D_{n,n^{\prime }}>c(\alpha )\sqrt{\frac{n+n^{\prime }}{nn^{\prime }}}$, where, for example, *α* = 0.05 corresponds to *c*(*α*) = 1.36. In our case, the critical value is *D_n,n′_* = 0.0192. In Tables 1 and 2, we show the values of the test statistic *D_n,n′_*, for different values of the number of quadrature points *Q* and orders of polynomial expansion *P* = *Q* − 1. We consider the root-MUSIC algorithm and the KR-root-MUSIC algorithm, respectively The two-sample KS test accepts, with a confidence of 95%, that, for the root-MUSIC algorithm and for *P* = *Q* − 1 = 4, the SCM samples and the MC samples have the same distribution. We also show the time necessary to obtain the cdf using the SCM method. Note that the simulation time to obtain the samples for the SCM algorithm is a factor more than 100 smaller than the simulation time to calculate the samples obtained by the MC method.

**Figure 9.**
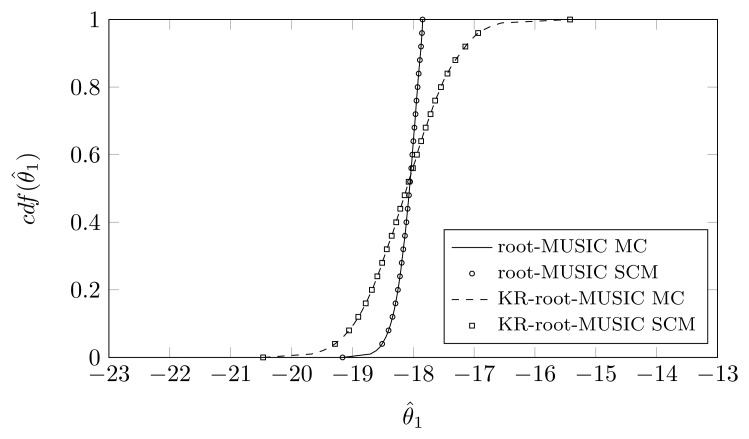
*P* = *Q* − 1 = 8, the cdf of *θ̂*_1_ when the first sensor element is displaced in the *x*-direction, *σ_pl_* = 0.12, true DOA = −18° and (*N*, *K*) = (4, 3).

**Figure 10.**
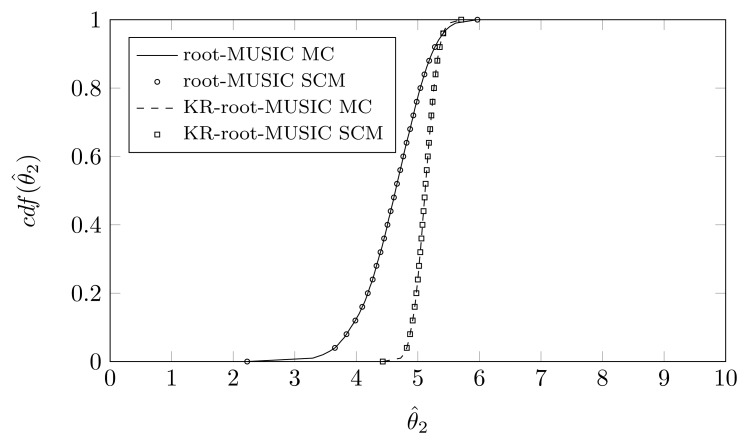
*P* = *Q* − 1 = 8, the cdf of *θ̂*_2_ when the first sensor element is displaced in the *x*-direction, *σ_pl_* = 0.12, true DOA = 5° and (*N*, *K*) = (4, 3).

**Figure 11.**
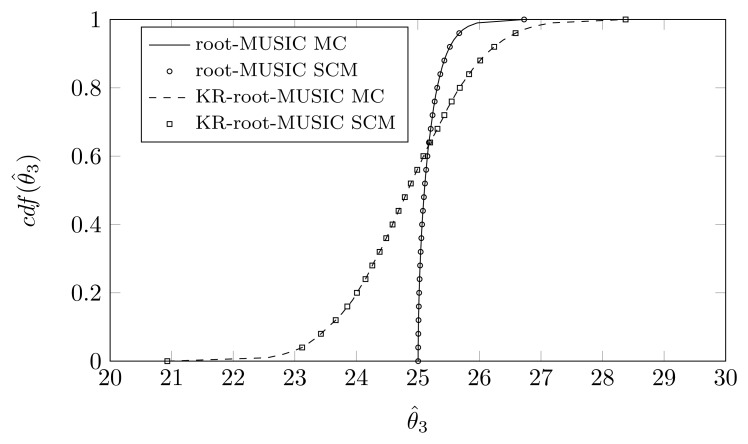
*P* = *Q* − 1 = 8, the cdf of *θ̂*_3_ when the first sensor element is displaced in the *x*-direction, *σ_pl_* = 0.12, true DOA = 25° and (*N*, *K*) = (4, 3).

For the KR-root-MUSIC algorithm, both empirical distributions are the same for *P* = *Q* − 1 = 4. In Figures 9, 10 and 11, we have plotted the cdfs both obtained by the SCM method (with *Q* = 9 quadrature points and a polynomial expansion of order *P* = 8) and the MC method. We observe that, even for a small number of quadrature points, we see almost no difference between the curves obtained by the SCM theory and the curves obtained by MC simulation, as indicated by the true sample KS test. Moreover, for the first and third estimated DOA, the root-MUSIC algorithm estimates the DOA very accurately. For the estimation of the second DOA, however, the KR-root-MUSIC algorithm performs better.

### 5.4. Two-Dimensional Displacement of One Sensor Element

In this section, we assume that the first sensor element is displaced by $\vec{r_{h}}=\left[e_{x}^{\text{rel}}d,e_{y}^{\text{rel}}d\right]$(Figure 1). The relative displacements in both the *x*-direction $e_{x}^{\text{rel}}$ and *y*-direction $e_{y}^{\text{rel}}$ are independently Gaussian distributed, with zero mean and dispersion *σ_pl_*. This means that the relative length of the displacement vector is Rayleigh distributed with mean $\sigma_{pl}\sqrt{\frac{\pi }{2}}$ and variance $\frac{4-\pi }{2}\sigma_{pl}^{2}$ and that the argument of the displacement vector is uniformly distributed in [−*π*, *π*].

For the same impinging signal as in Section 5.3, we have plotted the cdfs of the different estimated DOAs, for *σ_pl_* = 0.12. We have carried out an MC simulation with 10,000 realizations. The simulation time is about 128 s for the root-MUSIC algorithm and 157 s for the KR-root-MUSIC algorithm. We also apply the two-dimensional GPoC expansion (11) with degree *P* in both variables to obtain the cdfs for the DOAs. We calculate the weight coefficients using *Q*_1_ = *Q*_2_ = *P* − 1 quadrature points in the *x*- and *y*-directions using Equation (13). Tables 3 and 4 show the KS test to compare the samples obtained by the SCM method and the MC method. Furthermore, we compare this with the two-dimensional SCM theory that relies on the cubature formulas with *Q* = 44, *Q* = 99 and *Q* = 172, respectively, and with polynomial expansions of order *P* = 7, *P* = 10 and *P* = 15, respectively. The measured value of the KS test, which compares the samples of the MC method with those obtained with the cubature formulas, is also presented in Tables 3 and 4. Furthermore, the simulation times are shown in the last column of the tables. We observe that now, for the root-MUSIC algorithm, we need 14 quadrature points in each direction to satisfy the null hypothesis of the KS test (*α* = 0.05). Using cubature formulas, only for the estimates of the third DOA, 172 quadrature points are insufficient to satisfy the null hypothesis. For the KR-root-MUSIC algorithm, four quadrature points in each direction are sufficient. If we use cubature formulas, *Q* = 44 quadrature points are sufficient to satisfy the null hypothesis. We also observe that, using the SCM theory, the simulation time is reduced by a factor of about 20, and using the cubature formulas, we can reduce the simulation time by a factor of 56.

**Figure 12.**
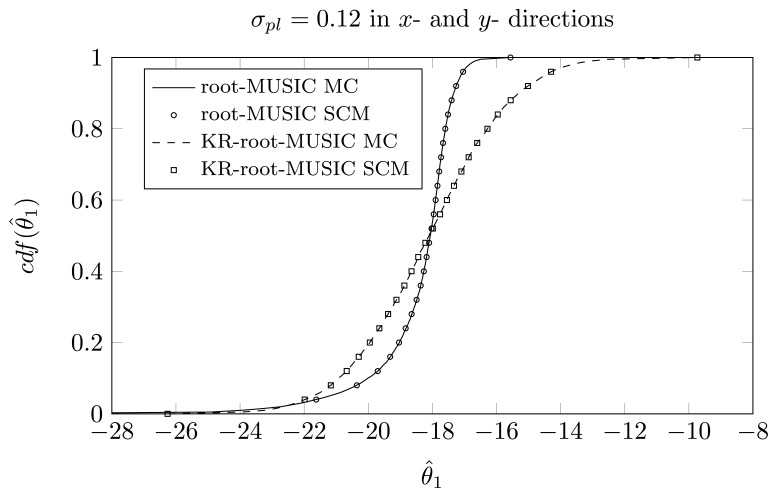
*P* = *Q* − 1 = 13, the cdf of *θ̂*_1_ for a two-dimensional displacement of first sensor element, true DOAs = [−18°, 5°, 25°] and (*N*, *K*) = (4, 3).

**Figure 13.**
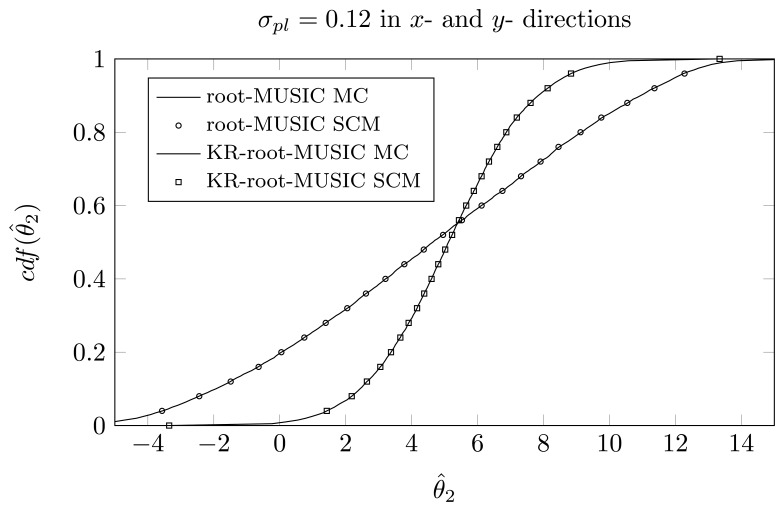
*P* = *Q* − 1 = 13, the cdf of *θ̂*_2_ for a two-dimensional displacement of first sensor element, true DOAs = [−18°, 5°, 25°] and (*N*, *K*) = (4, 3).

**Figure 14.**
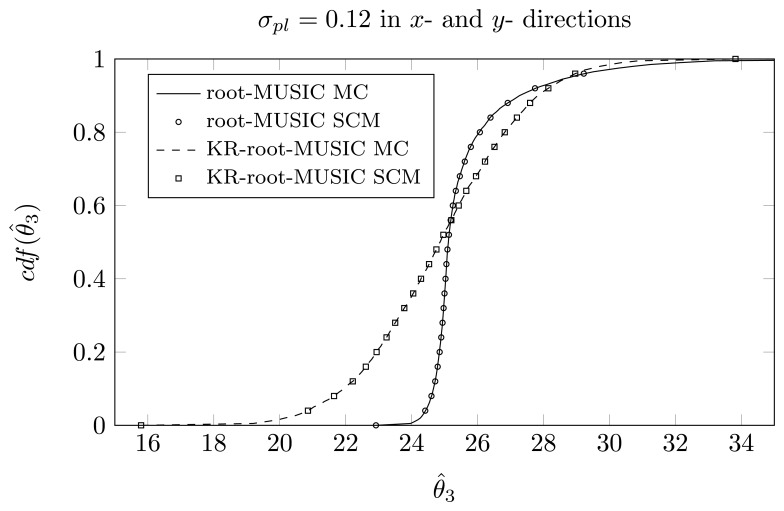
*P* = *Q* − 1 = 13, the cdf of *θ̂_3_* for a two-dimensional displacement of first sensor element, true DOAs = [−18°, 5°, 25°] and (*N*, *K*) = (4, 3).

In Figures 12, 13 and 14, we have plotted the empirical cdf for the MC simulation and for the SCM method *P* = *Q* − 1 = 13. We observe that both curves almost coincide.

**Figure 15.**
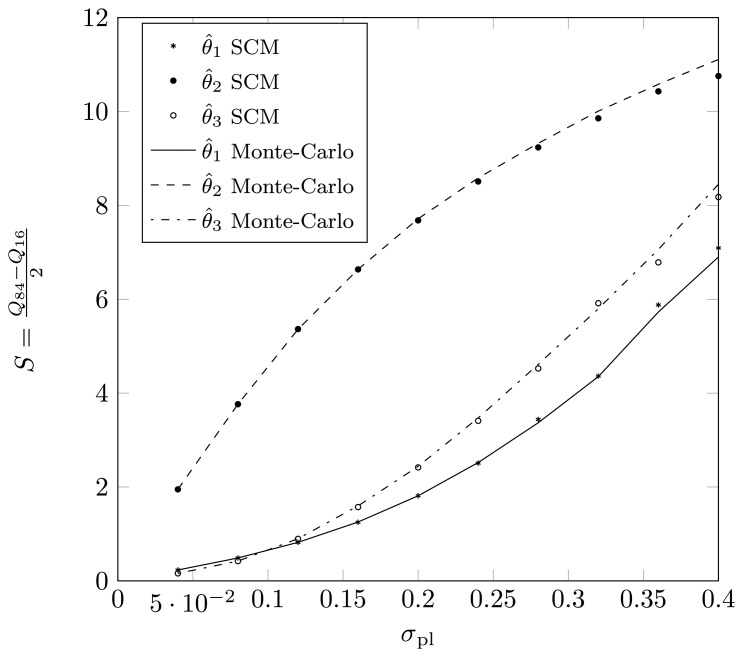
(*Q*_84_ − *Q*_16_)/2 as a function of *σ_pl_* for a two-dimensional displacement of the sensor element and when applying root-MUSIC, true DOAs = [−18°, 5°, 25°] and (*N*,* K*) = (4,3).

**Figure 16.**
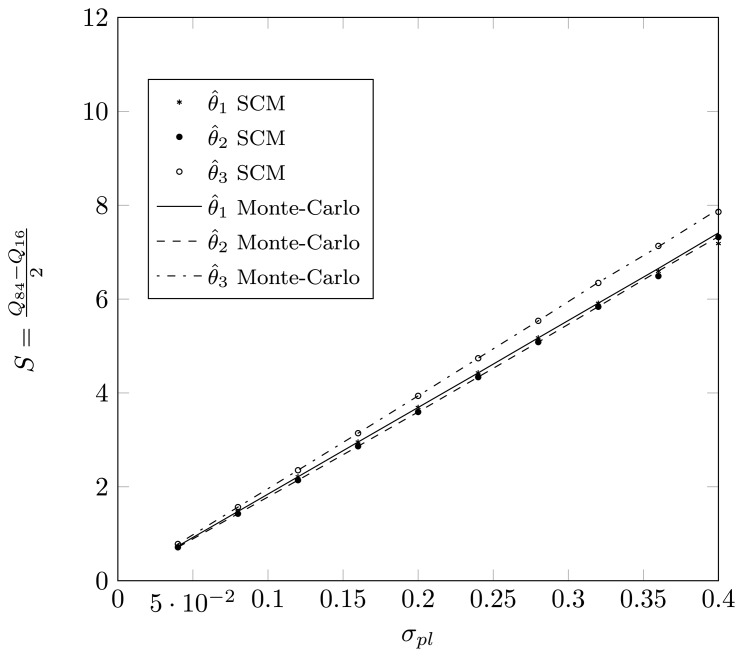
$\frac{Q_{84}-Q_{16}}{2}$ as a function of *σ_pl_* for a two-dimensional displacement of the sensor element and when applying KR-root-MUSIC, true DOAs = [−18°, 5°, 25°] and (*N*, *K*) = (4,3).

Define *Q_p_* as the *p*-th percentile of the estimated DOA, meaning that *p*% of the estimates for the true DOA are below *Q_p_*. Define $S=\frac{Q_{84}-Q_{16}}{2}$, such that 68% of the estimates lie in an interval of length 2*S* around the true DOA. For a Gaussian distribution, the value *S* will correspond to the dispersion. In Figures 15 and 16, we have plotted the value *S* as a function of *σ_pl_* for a two-dimensional displacement of the first sensor element. Again, we observe that the SCM method provides a good approximation for the estimates, both for the root-MUSIC algorithm as for the KR-root-MUSIC algorithm. Observe that for values *σ_pl_* less than 0.4, the root-MUSIC yields better estimates for the first and third DOA than the KR-root-MUSIC algorithm. However, the KR-root-MUSIC estimates the second DOA better. We furthermore observe that, for the KR-root-MUSIC algorithm, the *S*-values are straight lines as a function Of *σ_pl_*.

**Figure 17.**
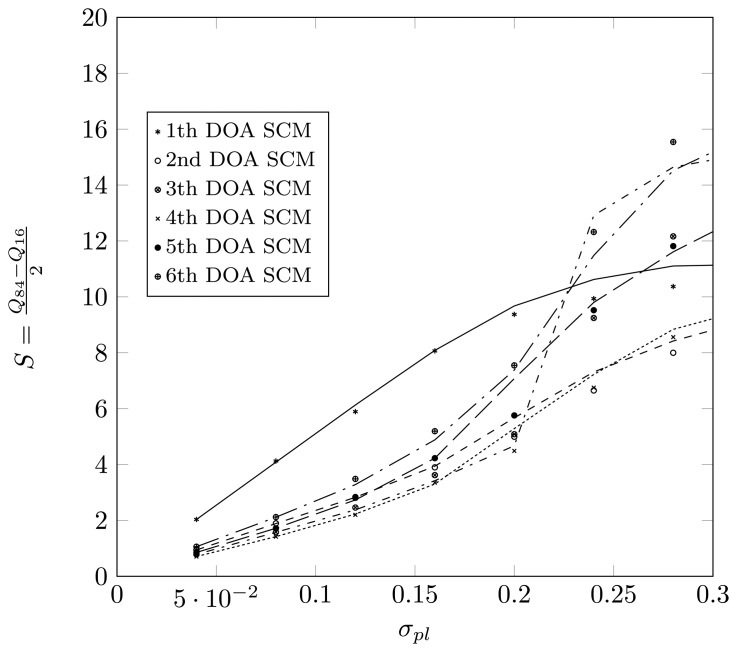
$\frac{Q_{84}-Q_{16}}{2}$ as a function of *σ_pl_* for a two-dimensional displacement of the sensor element and when applying KR-root-MUSIC, true DOAs = [−65°, −40°, −20°, 10°, 25°, 42°] and (*N*, *K*) = (4,6).

In Figure 17, we consider the (*N*,*K*) = (4,6) case with a displacement of the first sensor element and with the KR-root-MUSIC algorithm. The true DOAs to be estimated are [−65°, −40°, −20°, 10°, 25°, 42°]. Observe that, with the same array configuration as in Figure 16, we estimate twice as many DOAs. Looking at the $S=\frac{Q_{84}-Q_{16}}{2}$, we observe that the KR-root-MUSIC algorithm finds the first estimate for *θ̂*_1_ = −65° with a large error, even for small values of *σ_pl_*. The estimation error for the remainder of the DOAs starts to increase dramatically for values of *σ_pl_* larger than 0.2.

## 6. Conclusions

In this paper, we have investigated the effect of a displacement of one of the sensor elements. We have applied the SCM formalism to efficiently predict the cdfs of the estimated DOAs, in the case that the relative displacement is Gaussian distributed. Comparison of the SCM method to the Monte-Carlo method demonstrates that SCM is an accurate method to predict the cdfs of the DOAs. For the one-dimensional case, the SCM method decreases the simulation time by a factor of more than 100. For the two-dimensional case and using cubature formulas, we can reduce the simulation time by a factor of 56. In the future, we will extend the applied method to array configurations in which more sensor elements are displaced. Moreover, the SCM will be applied to other DOA estimation algorithms and for other array configurations. The flexibility and non-intrusive character of the proposed method only requires that DOA-estimates need to be computed in the quadrature points, by use of the appropriate algorithm.

## Figures and Tables

**Table 1.** The measured value *D_n,n^′^_* of the Kolmogorov–Smirnov (KS) test (critical value = 0.0192), one-dimensional shift along the *x*-axis of first sensor element, root-MUSIC algorithm, true DOAs = [−18°, 5°, 25°]; the last column is the simulation time.

root-MUSIC, *σ_pl_* = 0.12					
*D_n,n′_* for					
*P*	*Q*	*θ*_1_	*θ*_2_	*θ*_3_	time
4	5	0.0088	0.0004	0.0190	0.16 s
8	9	0.0002	0.0002	0.0006	0.17 s
12	13	0.0002	0.0001	0.0005	0.25 s
16	17	0.0001	0.0001	0.0002	0.39 s

**Table 2.** The measured value *D_n,n^′^_* of the KS test (critical value = 0.0192), one-dimensional shift along the *x*-axis of first sensor element, KR-root-MUSIC algorithm, true DOAs = [−18°, 5°, 25°]; the last column is the simulation time.

KR-root-MUSIC, *σ_pl_* = 0.12					
*D_n,n′_* for					
*P*	*Q*	*θ*_1_	*θ*_2_	*θ*_3_	time
4	5	0.0002	0.0001	0.0003	0.09 s
8	9	0.0001	0.0001	0.0001	0.33 s
12	13	0.0001	0.0001	0.0001	0.36 s
16	17	0.0001	0.0001	0.0001	0.55 s

**Table 3.** The measured value *D_n,n′_* of the KS test (critical value = 0.0192), two-dimensional displacement of first sensor element, root-MUSIC, true DOAs = [−18°, 5°, 25°]; upper part of table: DOAs obtained by the two-dimensional stochastic collocation method (SCM) method Equation (13); bottom part of table: DOAs obtained by cubature formulas (16); the last column is the simulation time.

root-MUSIC, *σ_pl_* = 0.12					
*D_n,n′_* for					
*P*_1_ = *P*_2_	*Q*_1_ = *Q*_2_	*θ*_1_	*θ*_2_	*θ*_3_	time
7	8	0.0251	0.0039	0.0731	4.14 s
10	11	0.0102	0.0025	0.0255	5.63 s
13	14	0.0038	0.0033	0.0112	6.40 s
*P*	*Q*	*θ*_1_	*θ*_2_	*θ*_3_	time
7	44	0.0134	0.0052	0.0486	0.76 s
10	99	0.0061	0.0021	0.0309	1.54 s
15	172	0.0044	0.0021	0.0219	2.22 s

**Table 4.** The measured value *D_n,n^′^_* of the KS test (critical value = 0.0192), KR-root-MUSIC, true DOAs = [−18°, 5°, 25°]; upper part of table: DOAs obtained by two-dimensional SCM method Equation (13); bottom part of table: DOAs obtained by cubature formulas (16); the last column is the simulation time.

KR-root -MUSIC, *σ_pl_* = 0. 12					
*D_n,n′_* for					
*P*_1_ = *P*_2_	*Q*_1_ = *Q*_2_	*θ*_1_	*θ*_2_	*θ*_3_	time
7	8	0.0002	0.0002	0.0002	4.74 s
10	11	0.0001	0.0002	0.0001	6.01s
13	14	0.0001	0.0001	0.0001	7.33 s
*P*	*Q*	*θ*_1_	*θ*_2_	*θ*_3_	time
7	44	0.0002	0.0002	0.0002	0.83 s
10	99	0.0003	0.0003	0.0004	1.62 s
